# Basal Cell Carcinoma in Patients over 80 Years Presenting for Surgical Excision: Clinical Characteristics and Surgical Outcomes

**DOI:** 10.3390/curroncol32030120

**Published:** 2025-02-21

**Authors:** Konstantinos Seretis, Nikos Bounas, Erasmia Rapti, Evangeli Lampri, Vasilios Moschovos, Efstathios G. Lykoudis

**Affiliations:** 1 Department of Plastic Surgery, Medical School, University of Ioannina, 45110 Ioannina, Greece; bounasnikos@gmail.com (N.B.); eri.rapti99@gmail.com (E.R.); billmosh@icloud.com (V.M.); elykoudi@uoi.gr (E.G.L.); 2 Department of Pathology, Medical School, University of Ioannina, 45110 Ioannina, Greece; elampri@uoi.gr

**Keywords:** basal cell carcinoma, keratinocyte, surgical excision, octogenarian, treatment, recurrence risk, NCCN

## Abstract

**Background.** Complete basal cell carcinoma (BCC) excision remains the most common treatment modality. However, its clinical characteristics and the surgical outcomes achieved in patients over 80 years—often with several medical comorbidities and potentially limited life expectancy—have not been thoroughly examined. This clinical study aims to investigate tumor-specific characteristics and surgical outcomes following surgical treatment of BCC in older individuals. **Methods.** An observational cohort study based on a prospectively maintained database was conducted in a tertiary center using a predetermined protocol. Patients who underwent BCC surgery between January 2010 and September 2024 were included and grouped by age under or over 80 years. The inclusion criterion was a histologically confirmed BCC, while patients with syndromes predisposing BCC development were excluded. **Results.** Among the 1396 biopsy-proven BCCs, 35% of the patients were older than 80 years. No significant differences were observed in their baseline characteristics. The pathogenic capacity was greater in elderly patients, who exhibited higher rates of multiple and concurrent skin cancers, larger BCC diameters, and routine involvement in high-risk areas. More lesions were classified as high-risk for recurrence, and the surgical treatment was accompanied by a higher frequency of positive or close margins, high-grade subtypes, and perineural invasion. Logistic regression of 1150 BCCs revealed that age > 80, advanced TNM stage, and margin status robustly predict high-risk histology and high NCCN risk of tumor recurrence. **Conclusions.** This study highlights that BCC in the elderly population tends to present with a more aggressive tumor status, based on the key clinical and pathology features. These findings underscore the need for tailored surgical strategies in this population.

## 1. Introduction

Basal cell carcinoma (BCC) remains the most common malignancy worldwide, demonstrating a progressively increasing annual incidence rate [1,2,3]. The prevalence of BCC is well-documented to increase with age [2,4,5,6]. At the same time, there has been a notable increase in the global geriatric population in recent years [7,8]. Despite the significant impact of BCC on a substantial portion of the population, research is impeded by its exclusion from national cancer surveillance systems. This difficulty is further compounded in the elderly population due to insufficient research and case documentation within this demographic [9].

Ultraviolet radiation (UVR) is directly linked to the development of BCC, which explains its higher prevalence on the face and the neck and the increased frequency with advancing age, with the nodular subtype being the most frequently encountered [4,6,10,11].

BCC is typically locally invasive, but surgical excision with adequate margins can achieve high cure rates, usually ensuring satisfactory cosmetic and functional outcomes, and thus, age alone should not be considered a contraindication for treating skin cancers in elderly patients [12]. However, its high prevalence results in a significant economic burden on healthcare systems worldwide. It is considered the fifth most costly malignancy after prostate, lung, colon, and breast cancer [5,13,14].

The limited research data thus far, combined with the difficulties in managing elderly patients and the rather indolent nature of this tumor, underscores the urgent need for a better understanding and further investigation of BCC in older patients. The aim of this study was to elucidate the clinical, surgical, and histopathological features of BCC in patients aged 80 years and over, in order to provide evidence for fine-tuning the relevant diagnostic and treatment protocols.

## 2. Materials and Methods

A retrospective observational cohort study was conducted at the Plastic Surgery Department of a tertiary referral hospital using a predetermined protocol, which conformed to the ethical guidelines of the 1975 Declaration of Helsinki, was approved by the local ethics committee (17/22-9-21, s.10), and adhered to the STROBE statement for cohort studies [15].

**Figure 1:**
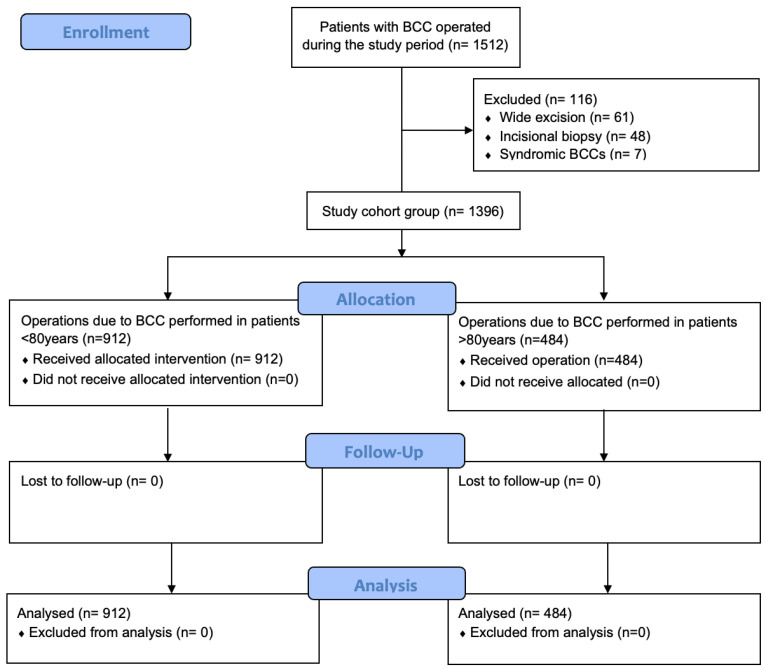
Study flow diagram of BCC excision in patients >80 years and <80 years old; n = number of operated BCC cases.

All patients who underwent excision of a skin tumor at the department between January 2010 and September 2024 were identified. Patients were included in the study if BCC was histologically confirmed. Records from patients with a syndrome associated with predisposition to BCC (i.e., Gorlin, xeroderma pigmentosum, Rombo, etc.), with incisional biopsy or wide excision following the biopsy result were excluded to ensure the study focused on sporadic cases (Figure 1). These syndromes involve distinct genetic mechanisms, higher tumor burdens, and unique treatment approaches, which could confound the findings and reduce the study’s relevance for the broader elderly population. The cohort was divided into two groups based on the patient’s age at the time of surgical treatment. Group A included patients less than 80 years old, while Group B included the older cohort. Postoperatively, all the patients were followed-up at regular intervals in the first year, and at the patients’ discretion in the long term. Relapse was diagnosed either at the follow-up visits or following clinical examination and confirmed by the biopsy results.

A prospectively maintained clinical database and the pathology reports were used to collect demographics, clinical, surgical, and pathological parameters of the study population. The outcomes of interest were age and sex of the patient, BCC location, histological subtype, largest diameter and depth (pathologically measured), NCCN clinical and pathological risk factors for recurrence, surgical margins and if complete excision was achieved, and recurrence.

### Statistical Analysis Methods

The null hypothesis was that the two groups have similar patient and tumor characteristics. Kolmogorov–Smirnov test was used to assess normality of distribution. Continuous variables were compared with an unpaired *t*-test or Mann–Whitney U test according to their distribution, whereas the chi-squared test was used for categorical variables. All statistical calculations were based on a two-sided hypothesis and considered statistically significant for *p*-values below 0.05.

Multivariate logistic regression analysis was performed to predict BCC aggressiveness in the study population. Firstly, we carefully defined and considered both the outcome variables and the independent variables included in the stepwise logistic regression models. The two primary outcomes were the high-risk histological subtype, representing the aggressive nature of the tumor based on the established histopathological criteria, and the high NCCN recurrence risk, indicating the likelihood of tumor recurrence according to the NCCN guidelines. The independent variables considered in both models included demographic factors (age, sex), tumor characteristics (e.g., TNM T stage, location, depth of invasion, perineural involvement), surgical factors (e.g., surgical margin status), and clinical history (e.g., prior recurrence). By assessing the direction (positive or negative) and strength of these associations, we included only the variables that demonstrated statistically significant associations with the outcomes. The data were analyzed using SPSS (IBM SPSS Statistics for Macintosh, Version 28).

## 3. Results

In total, 1396 histologically confirmed BCCs in 1162 patients were included in the study.

The characteristics and clinical course of the two groups are depicted in Table 1. Sixty percent of the tumors were identified in males, and 40% in females. The mean age was 71.86 ± 13.54 years, ranging from 27 to 100 years. While most patients were treated for one BCC (75.1%), one quarter of the patients were treated for more tumors concomitantly. Similarly, only 6.45% of the patients were managed surgically, apart from the BCC, for other skin cancers, mostly SCC (squamous cell carcinoma) or Bowen’s disease. The mean pathological tumor diameter was 11.92 ± 9.23 mm (range: 1–90 mm), with 74.7% of the tumors categorized histopathologically as T1 lesions.

Based on the recent NCCN guidelines, all risk factors of recurrence were examined [16]. Most of the tumors (1221, 87.5%) were located on the face, and thus were considered high-risk for recurrence (Table 1). The trunk was the second most frequent site of origin, representing, though, only 6.8% of the cases. No cases of prior radiotherapy were identified, while a minority of the patients were identified as immunocompromised (5%, *p* < 0.05 between the groups).

Based on the histopathological subtype, 59.1% of the cases were categorized as high-risk for BCC recurrence (Supplementary Materials, Table S1) [16].

Moreover, perineural invasion was identified in 65 cases (4.7%), while deep invasion, as defined in the NCCN guidelines for cutaneous SCC, was identified in 149 cases (10.7%, range: 0.1–15 mm) (Table 1) [16]. Overall, 93.8% of the cases presented with high-risk factors of recurrence, either clinical or histopathological.

Surgical excision was proven effective in the pathology report in almost 80% of the cases (Supplementary Materials, Table S2). However, 22.4% of the excisions had negative peripheral or deep margins of less than 1 mm. In this cohort with 15 years of follow-up, the relapse rate was measured as low (6.9%, 97 cases) (Supplementary Materials, Table S2).

The defects were reconstructed either with primary repair, a skin graft, or a flap. Based on the NCCN guidelines, radiotherapy was administered when required, which was performed uneventfully. A low rate of postoperative complications was reported, mostly wound healing problems (2.3%), infection (1.6%), and hematoma (0.7%). No major complications (death, functional loss) were recorded. Further surgical treatment due to wound healing problems and/or skin graft or flap necrosis was required in 1.2% of the cases. No statistically significant difference was revealed between the two age cohorts.

The findings of the comparison between the two BCC groups are summarized in Table 1. No statistically significant difference between the groups was identified in terms of sex of the patients, history of immunosuppression or prior radiation therapy, and anatomic distribution (face vs. other locations). However, a significant difference was revealed in the number of BCCs treated per patient, concomitant treatment of other skin cancers, and tumor size, as measured pathologically or defined by TNM. In addition, the older group of patients presented with more aggressive pathological features, as defined by the BCC subtype, perineural involvement, deep tumor invasion, and overall NCCN risk of recurrence. Although similar rates of negative margins were achieved (77.7% vs. 73.7% in the older cohort, *p* < 0.05), the subgroup analysis of clearance rates in the two cohorts was significant (favoring the younger cohort) when the close margins were measured separately (*p* < 0.001). Relapse rates were found to be borderline insignificant.

Regression analysis was used to examine aggressiveness in this cohort, using the histological subtype (high- or low-risk) and the NCCN risk of recurrence as dependent variables. In the first analysis, 1150 BCCs with complete data were examined. The independent variables in this model were the following categorical variables: sex, age (over/under 80 years), recurrence, location, TNM T group (T1–T4), NCCN risk, surgical margin achieved, depth of invasion (below/above 6 mm), and perineural involvement. The stepwise logistic regression analysis showed that the model statistically significantly predicts the dependent variable (Supplementary Materials, Table S3A–D). In particular, the predictive model for a high-risk histological subtype includes the following parameters: age (over 80 years), TNM T (tumor diameter), margin achieved, and perineural involvement.

The regression analysis for the NCCN risk of recurrence (high/low) of the 1150 BCCs examined the following categorical variables: sex, age (over/under 80 years), histological subtype aggressiveness (high/low), location, TNM T group (T1–T4), surgical margin achieved (positive/negative), depth of invasion (below/above 6 mm), recurrence, and perineural involvement. The multivariate logistic regression analysis showed that the model statistically significantly predicts the dependent variable (Supplementary Materials, Table S4A–D). The predictive model for a high risk of BCC recurrence, based on the NCCN guidelines, includes the following parameters: age (over 80 years), TNM T (tumor diameter), margin achieved, and recurrence.

## 4. Discussion

The ill-defined umbrella designation of non-melanoma skin cancer (NMSC) or the recently proposed more precise and reasonable terminology of keratinocyte skin cancer (KSC) encompasses the two most common types of skin cancer, namely, BCC and SCC [17]. The incidence of BCC is still increasing while the population is aging worldwide, with recent data showing a steady increase in people over 80 years of age [1,2,3,18]. Considering the well-known correlation between aging and skin cancer, this large observational comparative study aimed to clarify the disease potential of BCC in patients over 80 years old. Specifically, the pathogenic capacity of BCC was notably higher in the elderly group as these patients exhibited significantly elevated rates of simultaneous BCC lesions, concurrent skin cancers, or subsequent diagnosis of additional skin cancers during follow-up. From a clinical standpoint, they also presented with larger BCC tumor diameters and demonstrated a predilection for the high-risk areas such as the face. Evidently, more lesions were categorized as high-risk for recurrence based on the NCCN guidelines. The recurrence rates in the elderly group were found to be borderline insignificant, marking a trend easily comprehensible due to the higher-risk subtypes. As a result of this aggressive profile, achieving clear surgical margins often proved troublesome, with a higher proportion of patients yielding positive or close margins on biopsy specimens. Careful analysis of the pathology reports verified the aforementioned claims, revealing an increased frequency of high-grade subtypes along with perineural involvement and deep invasion. Predictive models, established via regression analysis, confirmed that age > 80 was a significant variable for determining a high-risk histologic subtype and BCC at high risk of recurrence, based on the NCCN guidelines. Overall, the older cohort of patients with BCC had an elevated NCCN recurrence risk.

The incidence of KSC increases with age, particularly in the head and neck regions, likely reflecting the cumulative effects of UV radiation, specifically acute exposure periods in an intermittent fashion due to modern lifestyle habits [19,20]. Other predisposing factors include Fitzpatrick phototype, family or personal history of skin cancer, and carcinogenic chemicals, such as arsenic [21,22]. It goes without saying that the decline in immunological surveillance consistently observed in older adults is also a key contributing factor in the intricate pathogenesis of BCC [10]. At this point, the observed increased incidence rates of BCC may also be attributed to the constant advances in medical screening protocols that enable more accurate and prompt diagnosis. Additionally, as previously discussed, the financial toll and the workload shouldered by the healthcare system for BCC treatment is substantial, with data from 2013 alone indicating that approximately $715 million dollars were spent on direct BCC care in Medicare beneficiaries [23]. To that end, successful public awareness campaigns have been formulated to inform the public, especially the elderly populations, further increasing the numbers of KSC-diagnosed patients.

The pathogenesis of BCC has been widely studied, and it is now acknowledged that dysregulation of the Hedgehog pathway (Hh) is directly linked with this malignancy [24,25]. The Hh signaling pathway is a crucial regulator of embryonic development and tissue homeostasis. It begins when Hedgehog ligands (e.g., Sonic Hedgehog, Desert Hedgehog, Indian Hedgehog) bind to transmembrane receptor Patched (PTCH1), relieving its inhibition of another protein called Smoothened (SMO) [24]. Activated SMO initiates an intracellular signaling cascade that leads to the activation of the Gli family of transcription factors (Gli1, Gli2, Gli3) [24]. These factors translocate to the nucleus and regulate the expression of target genes involved in cell proliferation, differentiation, and survival. Somatic mutations in PCTH1 are the most frequently observed, ranging between 11% and 75%, and are represented by non-synonymous mutations with a predominance of nonsense and splice site mutations throughout the entire length of the PTCH1 gene [26]. Approximately half of these mutations contain the “UV-signature” C→T and tandem CC→TT transitions, while other factors, such as oxidative stress, have also been implicated [27,28]. The second most frequent event associated with BCC pathogenesis is TP53 gene inactivation [29]. Likewise, most TP53 mutations in basal cell carcinoma are characterized by C to T transitions, with a high prevalence of CC to TT tandem mutations, strongly indicating UV-induced DNA damage, a claim supported by a study that proved that BCCs in sunscreen users exhibited a lower frequency of TP53 mutations compared to those in non-sunscreen users [30].

Obviously, it can be easily inferred that older individuals, who have experienced prolonged UV radiation exposure, are likely to show a higher incidence of BCC lesions. Recent research on BCC mutational mechanisms revealed that nascent BCC-like tumors can enter a dormant state after initial PTCH1 loss. However, tumors that acquire additional mutations can escape this dormancy and progress to more aggressive forms. This implies that while initial Hh pathway mutations are critical, the accumulation of further genetic alterations is necessary for aggressive tumor characteristics [26]. The elderly should, therefore, be considered extremely prone to this since amassed DNA damage over their longer lifespans, along with the telomere shortening phenomenon for which substantial evidence supports a role in impacting health and lifespan, is directly linked with cancer [31,32]. Several other factors may also be at play in the cellular microenvironment, such as the chronic inflammation induced by age, referred to as “inflammaging”, and the mitochondrial function decline, leading to an increased production of reactive oxygen species, promoting states of higher oxidative stress favoring tumorigenesis, as already discussed [33,34].

The evidence of the currently available medical literature is widely supportive of the aforementioned claims. Maisel-Campbell et al. reported in their multicenter prospective cohort study including 17,076 patients that among BCCs, tumors with aggressive histological subtypes were more common in the patients older than 85 years compared with the younger patients [35]. On the same page, our regression analysis model highlighted age > 80 as a significant predictor for encountering tumors with aggressive histologic traits. Additional predictive variables included tumor diameter and perineural involvement, both of which are regarded as textbook characteristics of histologic aggressivity, as well as positive surgical margins, an expected feature of the more aggressive tumor patterns and associated with lower rates of achieving clear surgical margins in the clinical setting. Asgari et al. conducted a large study on the population of California and managed to include a total of 147,093 patients presenting BCC lesions, observing that the increase in incidence of the malignancy was greatest among the patients 80 years or older for both sexes (annual percentage change (APC): 2.72; 95% CI (2.35–3.10) for female patients and APC, 1.67; 95% CI (1.35–1.99) for male patients) [3]. A retrospective study from Poland by Ciążyńska et al., involving 11,846 BCC cases, further supported the increased incidence of BCC in individuals over 75 years old [36]. The study also found that tumors were more commonly located in high-risk areas, such as the face, with a lower incidence observed in low-risk areas [36]. The results of our study correspond to those already discussed, which are denotative of highly aggressive tumors in the elderly apart from the increased incidence rates observed.

Our findings align with the existing literature, reinforcing the fact that older individuals typically present with larger, more invasive BCC tumors, often with a deeper microscopic invasion, visible macroscopic characteristics, and locally advanced stages [37,38,39]. Notably, these tumors exhibit more aggressive growth patterns and histological subtypes in the elderly [35]. Moreover, studies highlight an association between tumor location and histological subtype with patient age, where elderly patients frequently develop tumors in areas with a higher recurrence risk, such as the face and the extremities [37,40,41,42].

Although BCC incidence generally increases with age in both males and females, the majority of studies report a higher prevalence in men [3,6,11]. However, our study, consistent with some literature, found no significant sex-based difference in BCC incidence, especially in older populations where survival rates among elderly women might account for a more equal distribution [40,43]. While younger men show a well-documented predominance of BCC cases, sex disparity tends to balance out in the elderly [40].

The NCCN guidelines for BCC delineate the factors that place the patients in the high-risk category for recurrence and, thus, necessitate more aggressive treatment options [16]. This increased recurrence risk in the elderly may be closely tied to such factors as immune system decline and cumulative environmental damage over time, predisposing this age group to multiple BCC lesions [10,44]. However, the link between age and recurrence remains contentious: some research finds no age-related predictiveness for recurrence, whereas other studies suggest that advanced age may elevate the recurrence risk, especially in high-grade histological subtypes or cases of incomplete excision [10,37,45,46,47]. Our results suggest a positive correlation between age and the risk of recurrence, emphasizing the need for complete surgical resection with sufficient margins in elderly patients to mitigate this risk [48]. Our regression analysis model clearly indicated that age > 80 is a significant independent predictor for lesions classified as high-risk for recurrence, according to the NCCN guidelines. Additional predictive variables included tumor diameter and recurrent tumors, both of which are established among the NCCN criteria for high risk classification, as well as positive surgical margins, which are a clear predisposing situation for tumor recurrence. Age-related immunosenescence likely contributes to decreased anticancer responses after an incomplete excision, highlighting the critical role of thorough surgical removal, which is often associated with the need for complex reconstruction techniques [49,50].

Treatment of BCC in elderly patients poses unique challenges, with comorbidities, functional and nutritional status, and polypharmacy requiring careful consideration [6,10]. Our data and most studies indicate that age alone does not significantly impact postoperative complications and relapse rates, thus underscoring that age should not be the sole factor in skin cancer management [9,10,37,39]. Surgical excision remains the preferred treatment for BCC in older populations, given the aggressive nature of these tumors [35]. Without a prompt intervention, BCC can significantly affect the quality of life through increased morbidity, reinforcing the necessity of timely and effective treatment for elderly patients [18]. Recent advances in this field, such as Raman spectroscopy and intraoperative flow cytometry, seem to pave the way for a more accurate, direct intraoperative diagnosis and confirmation of complete excision of KSCs, which is particularly important in this older group of patients [51,52].

Among the strengths of this study, the large sample size obviously stands out, enhancing the accuracy and validity of the presented results. Patient records and follow-up data were extremely consistent, which allowed systematic data collection on multiple variables of interest, while simultaneously limiting potential losses of key information. The rigorous methodology employed is a key strength of this cohort study, utilizing evidence from a prospectively maintained database in the best possible manner. Thus, multiple outcomes of interest were thoroughly investigated, yielding statistical significance in an overwhelming percentage of cases, further supporting our conclusions. In addition, all the patients were treated by only three surgeons and at the same institution, mitigating any confounding factors associated with the operation or techniques applied.

On the other hand, our study is subject to limitations. The single-center study design and the inclusion of patients treated exclusively via surgery may have introduced selection bias in terms of aggressive BCC characteristics. However, the comparative study design limits the effect of the treatment modality, highlighting the different features of the two age groups. Furthermore, data from a prospectively maintained database do not ensure that all relevant confounders have been recorded or adequately controlled for, which could still introduce a degree of bias into the results. The two groups are not evenly distributed, with patients over 80 years accounting for only 35% of the total sample. Finally, the outcome on relapse rates observed between the two age groups cannot be interpreted with strong confidence due to the shorter follow-up of the older cohort, contributing to reduced data availability, and thus, this outcome was precluded from further analysis.

Given the increasing incidence of BCC among older adults, future prospective studies involving large patient cohorts are essential to establish specific assessments for older individuals with BCC. Studies should focus on both clinical and histopathological characteristics to advance our knowledge of the nature of these tumors in older patients. Moreover, subsequent research should concentrate on clarifying the prognostic factors associated with BCC recurrence and identifying the best practices for surgical intervention, particularly regarding complete resection with adequate margins [53]. Research should also focus on optimizing treatment modalities in the very elderly, particularly regarding the efficacy and safety of various interventions. Understanding the relationship between tumor characteristics, patient age, and health-related quality of life will be vital in developing comprehensive management plans that prioritize both clinical outcomes and patient well-being. The ultimate objective is to develop a personalized treatment approach and a cost-effective care pathway tailored to the needs of an aging population dealing with BCC.

## 5. Conclusions

In conclusion, this clinical study managed to pinpoint that the elderly(over 80 years) exhibit BCCs with a more clinically aggressive status based on the key clinical and pathological features, such as tumor dimensions, depth of invasion, and histological subtype. Surgical specialties dealing with skin cancer should remain vigilant in light of these data and devise proper, even more dynamic treatment plans for this age group in order to minimize morbidity and achieve higher cure rates. Further well-planned multicenter studies and meta-analyses need be conducted to cement these observations and ameliorate the existing treatment algorithms to better guide clinical decision-making to the benefit of the patients’ health. Future research should also explore the molecular and genetic factors contributing to the aggressive nature of BCC in elderly populations.

## Figures and Tables

**Table 1:** Clinicopathological characteristics of excised BCCs in patients >80 years and <80 years old.

Characteristic	<80 y Group n (%)	>80 y Group n (%)	*p*
Patient’s sex	912	484	
Male	555 (60.9%)	283 (58.5%)	0.39
Female	357 (39.1%)	201 (41.5%)	
Patient’s age at diagnosis (y)			
Range, mean ± SD, median	27–79, 64.94 ± 11.18, 68	80–100, 85.31 ± 4.09, 85	**<0.001**
Treated BCCs/patient			
1	717 (78.6%)	332 (68.6%)	
>1	195 (21.4%)	152 (31.4%)	**<0.001**
Treated BCC and other tumors/patient			
Yes	44 (4.8%)	46 (9.5%)	
No	868 (95.2%)	438 (90.5%)	**<0.001**
Tumor size (mm)			
Range, mean ± SD, median	1–90, 10.87 ± 8.80, 8	1–87, 13.90 ± 9.67, 12	**<0.001**
Tumor size (TNM)			
T1	718 (78.7)	325 (67.1%)	
T2	83 (9.1)	72 (14.9%)	**<0.001**
T3	110 (12.1)	87 (18.0%)	
T4	1 (0.1)	0 (0%)	
Location			
Face	782 (85.7)	439 (90.7%)	
Neck	30	12	
Hand	0	4	
Foot	0	1	
Pretibia	7	3	
Anogenital	1	2	**<0.003**
Trunk	77	18	
Extremities	15	5	
Perineural invasion (yes)	33 (3.6%)	32 (6.6%)	**0.016**
Deep invasion (>6 mm)	89 (9.8%)	60 (12.4%)	**<0.001**
NCCN risk			
Low	73	14	**<0.001**
High	839	470	

Note: y: years, n: number of cases, SD: standard deviation. *p*-values are bold when *p* < 0.05. T1: tumor ≤ 2 cm in the greatest dimension. T2: tumor > 2 cm but ≤ 4 cm in the greatest dimension. T3: tumor >4 cm, minor bone erosion, perineural invasion, or deep invasion. T4: tumor with gross cortical bone/marrow, skull base invasion, or skull base foramen involvement.

## Data Availability

The data are contained within the article or the Supplementary Materials.

## Supplementary Materials

The following supporting information can be downloaded at: https://www.mdpi.com/article/10.3390/curroncol32030120/s1, Table S1. BCC histopathological subtypes in patients >80 years and <80 years old. Table S2. Efficacy of surgical treatment and relapse rates of BCC in patients >80 years and <80 years old. Table S3. Multinomial logistic regression analysis for high-risk histological subtype. Table S4. Multinomial logistic regression analysis for NCCN high-risk for recurrence.
