# Supplementary material for: Basal Cell Carcinoma in Patients over 80 Years Presenting for Surgical Excision: Clinical Characteristics and Surgical Outcomes
Source: Curr Oncol. 2025 Feb 21;32(3):120. doi: 10.3390/curroncol32030120 (PMC11940936; doi:10.3390/curroncol32030120)
Supplement: Supplementary file 1 [file curroncol-32-00120-s001.zip › curroncol-3434629-supplementary.pdf]

Table S1. BCC histopathological subtypes in patients >80 years and <80 years old.

**BCC Subtype**	**<80y Group n (%)**	**>80y Group n (%)**	**p**
**Low risk**	**328 (45.8%)**	**142 (32.7%)**	
Nodular	243	119	
Nodulocystic	9	2	
Superficial	59	16	
Infundibulocystic	17	5	
**High Risk**	**388 (54.2%)**	**292 (67.3%)**	**<0.001**
Basosquamous	178	152	
Mixed	140	90	
Infiltrative	44	36	
Micronodular	14	7	
Adenoid	8	6	
Morpheaform	4	1	
Not reported	196	50	

n: number of cases.

Table S2. Efficacy of surgical treatment and relapse rates of BCC in patients >80 years and <80 years old.

**Treatment efficacy**	**<80y Group n (%)**	**>80y Group n (%)**	**p**
**Tumor clearance (n)**			
Yes (%)	386 (50.6%)	212 (49.0%)	
Yes but <1mm (%)	206 (27.1%)	107 (24.7%)	**<0.001**
No (%)	170 (22.3%)	114 (26.3%)	
**Relapse (n)**			
No	857 (94.0%)	442 (86.8%)	0.076
Yes	55 (6.0%)	42 (13.2%)	

n: number of cases.

Table S3. Multinomial logistic regression analysis for high-risk histological subtype.

A.

Model Fitting Information

	Model Fitting Criteria	Likelihood Ratio Tests		
Model	-2 Log Likelihood	Chi-Square	df	Sig.
Intercept Only	234.767			
Final	110.669	124.098	9	<.001

B.

Goodness-of-Fit

	Chi-Square	df	Sig.
Pearson	28.552	28	.436
Deviance	31.549	28	.293

In Table 1A, the "**Sig.**" column that $p < .001$, means that the full model statistically significantly predicts the dependent variable better than the intercept-only model alone.

In Table 1B, the first row, labelled "**Pearson**", presents the Pearson chi-square statistic. A statistically significant result (i.e., $p < .05$) indicates that the model does not fit the data well. You can see from the table above that the *p*-value is .436 (from the "**Sig.**" column) and is, therefore, not statistically significant. Based on this measure, the model fits the data well.

C.

Likelihood Ratio Tests

	Model Fitting Criteria	Likelihood Ratio Tests		
Effect	-2 Log Likelihood of Reduced Model	Chi-Square	df	Sig.
Intercept	110.669[a]	.000	0	.
Age group 80	123.041	12.371	1	<.001
T (TNM)	151.603	40.934	4	<.001
Margin	123.485	12.815	3	.005
Perineural inv.	121.019	10.350	1	.001

The chi-square statistic is the difference in -2 log-likelihoods between the final model and a reduced model. The reduced model is formed by omitting an effect from the final model. The null hypothesis is that all parameters of that effect are 0.

a. This reduced model is equivalent to the final model because omitting the effect does not increase the degrees of freedom.

This table shows that the independent variables included in the model are statistically significant. This table is mostly useful for nominal independent variables because it is the only table that considers the overall effect of a nominal variable.

D.

Parameter Estimates

**RiskSubtype**[a]		B	Std. Error	Wald	df	Sig.	Exp(B)	95% Confidence Interval for Exp(B) Lower Bound	Upper Bound
1	Intercept	15.770	.256	3780.255	1	<.001			
	[80=OLD]	.465	.133	12.231	1	<.001	1.592	1.227	2.065
	[80=YOUNG]	0[b]	.	.	0	.	.	.	.
	[T=]	-16.763	.971	298.330	1	<.001	5.248E-8	7.833E-9	3.516E-7
	[T=1]	-16.005	.251	4056.482	1	<.001	1.120E-7	6.846E-8	1.833E-7
	[T=2]	-15.254	.308	2456.855	1	<.001	2.373E-7	1.298E-7	4.337E-7
	[T=3]	-14.762	.000	.	1	.	3.880E-7	3.880E-7	3.880E-7
	[T=4]	0[b]	.	.	0	.	.	.	.
	[Margin=]	.666	.262	6.483	1	.011	1.947	1.166	3.253
	[Margin=close]	.084	.156	.291	1	.590	1.088	.802	1.476
	[Margin= -]	.483	.174	7.704	1	.006	1.621	1.153	2.280
	[Margin= +]	0[b]	.	.	0	.	.	.	.
	[Perineural=Yes]	1.955	.759	6.628	1	.010	7.063	1.595	31.286
	[Perineural =No]	0[b]	.	.	0	.	.	.	.

a. The reference category is: 0 (low risk histological subtype).
b. This parameter is set to zero because it is redundant.

This table presents the parameter estimates (also known as the coefficients of the model). As there were two categories of the dependent variable (low/high risk histological subtype), you can see that there is one set of logistic regression coefficients, representing the comparison of high-risk subtype category to the reference category, low-risk subtype.

The statistically significant coefficients have a Sig. value (from the "**Sig.**" column) of <0.05.

Odds ratio for each variable is presented as Exp(B), with 95% confidence intervals reported in the last two columns.

Table S4. Multinomial logistic regression analysis for NCCN high-risk for recurrence.

A.

Model Fitting Information

Model	Model Fitting Criteria	Likelihood Ratio Tests		
	-2 Log Likelihood	Chi-Square	df	Sig.
Intercept Only	92.779			
Final	32.133	60.647	6	<.001

B.

Goodness-of-Fit

	Chi-Square	df	Sig.
Pearson	24.997	18	.125
Deviance	14.375	18	.704

The full model statistically significantly predicts the dependent variable better than the intercept-only model alone. Table 1B shows that the model fits the data well.

C.

Likelihood Ratio Tests

Effect	Model Fitting Criteria	Likelihood Ratio Tests		
	-2 Log Likelihood of Reduced Model	Chi-Square	df	Sig.
Intercept	32.133[a]	.000	0	.
Age group 80	42.294	10.161	1	.001
Recurrence	39.776	7.643	1	.006
T (TNM)	49.146	17.014	3	<.001
MARGIN	45.960	13.827	1	<.001

The chi-square statistic is the difference in -2 log-likelihoods between the final model and a reduced model. The reduced model is formed by omitting an effect from the final model. The null hypothesis is that all parameters of that effect are 0.

a. This reduced model is equivalent to the final model because omitting the effect does not increase the degrees of freedom.

This table shows only the independent variables included in the model, which are statistically significant.

D.

Parameter Estimates

NCCN RISK[a]		B	Std. Error	Wald	df	Sig.	Exp(B)	95% Confidence Interval for Exp(B) Lower Bound	Upper Bound
1	Intercept	17.809	.897	393.900	1	<.001			
	[80=OLD]	.957	.327	8.581	1	.003	2.603	1.372	4.936
	[80=YOUNG]	0[b]	.	.	0	.	.	.	.
	[Recur=Yes]	14.475	682.934	.000	1	.983	1933506.037	.000	.[c]
	[Recur=No]	0[b]	.	.	0	.	.	.	.
	[T=1]	-13.875	.602	530.876	1	<.001	9.421E-7	2.894E-7	3.067E-6
	[T=2]	-11.495	1.165	97.421	1	<.001	1.018E-5	1.039E-6	9.982E-5
	[T=3]	-12.665	.000	.	1	.	3.159E-6	3.159E-6	3.159E-6
	[T=4]	0[b]	.	.	0	.	.	.	.
	[MARGIN= -]	-1.970	.725	7.389	1	.007	.139	.034	.577
	[MARGIN= +]	0[b]	.	.	0	.	.	.	.

a. The reference category is: 0 (low risk for recurrence NCCN).
b. This parameter is set to zero because it is redundant.
c. Floating point overflow occurred while computing this statistic. Its value is therefore set to system missing.

This table presents the coefficients of the model. As there were two categories of the dependent variable (NCCN low/high risk for recurrence), you can see that there is one set of logistic regression coefficients, representing the comparison of high-risk category to the reference category, NCCN low-risk for recurrence.

The statistically significant coefficients have a Sig. value (from the "**Sig.**" column) of <0.05.

Odds ratio for each variable is presented as Exp(B), with 95% confidence intervals reported in the last two columns.
